# The Relationship Between the Type of Crime and Drugs in Addicted Prisoners in Zahedan Central Prison

**DOI:** 10.5812/ijhrba.13977

**Published:** 2013-12-22

**Authors:** Raheleh Rafaiee, Saeede Olyaee, Alireza Sargolzaiee

**Affiliations:** 1 Shahroud University of Medical Sciences, Shahroud, IR Iran; 2 Zahedan University of Medical Sciences, Zahedan, IR Iran; 3 Zahedan Central Prison, Zahedan, IR Iran

**Keywords:** Behavior, Addictive, Crime, Prisons

There is a close relationship between drug abuse and crime. Drug abusers commit crimes to pay for their drugs and this inflicts damages to the society. Moreover, many criminals are under the influence of drugs while committing crimes. Drug trafficking is another outcome of drug abuse (1).

According to a research in the United States in 2010, 70% of male prisoners were drug abusers which is significant compared to the 11.2% rate of drug abuse in the entire male population (2). Alcohol has the highest relationship with aggressive crimes. According to reports, criminals who had abused drugs and alcohol simultaneously committed 21.4% of aggressive crimes. Among individuals who were arrested in Australia during 2004, 82% had a history of drug abuse, 69% had abused drugs at least 6 months before their arrest and 62% frequently abused drugs (3).

This cross sectional descriptive analytical study was designed and carried out to determine the relationship between the type of crime and the drug abused by addicted prisoners. The statistical population consisted of all 923 addicted male prisoners in Zahedan central prison who were under methadone maintenance treatment in prison. They were selected by census. The information collection tool was via a questionnaire and an interview. Research data was analyzed with the SPSS 19 software using descriptive indicators of statistical analysis. Apart from demographic factors and the individual’s addiction history, the type of crime was investigated as well.

The average age of the prisoners was 33.84 years, the average age of addiction to drugs was 20.77, involvement duration was 11.94 years, 69% were married and 31% were single. Abused substances were Iranian crack, opium syrup, methamphetamine, heroin, psychotropic pills and cannabis. The crimes of opiate drug abusers (opium syrup, opium, heroin and Iranian crack) in order of priority included: drug related crimes (crack 63.7%, heroin 52.4%, opium 43.8%, opium syrup 40.5%), robbery (crack 63.3%, heroin 52.4%, opium syrup 22%, opium 21.6%), murder (opium 21.6%, crack 18.30%, opium syrup 14.5%, heroin 9.7%), armed robbery (heroin 25%, opium syrup 22%, crack 21.1%, opium 12.4%) and kidnapping (opium 4.3%, opium syrup 4.2%, crack 2.6%, heroin 2.4%). The crimes of hallucinogenic drugs (hashish, psychotropic pills, glass or methamphetamine) included: robbery (glass 53.2%, pills 36.6%), drug-related crimes (hashish 33.3%, glass 21.6%, pills 9.1%), armed robbery (hashish 33.3%, pills 9.1%, glass 5.4%) and murder (pills 18.2%, hashish 16.7%, glass 8.8%).

There was a direct relationship between the level of drug abuse and the type of drug and committed crimes. With increased drug abuse, delinquency rate and its intensity increased. Addicts are forced to commit crime to acquire drugs. Addicts are not hired. As a result, they don’t have an income to fulfill their needs. That’s why they turn to illegal activities such as smuggling, drug dealing, theft and prostitution. Thus the cycle of poverty, addiction, and crime is repeated. The necessity of developing preventive strategies is felt more than before.
